# Association Between Thyrotoxicosis and Cerebral Venous Thrombosis

**DOI:** 10.3390/jcm13216547

**Published:** 2024-10-31

**Authors:** Margherita Paccagnella, Anna Pizzo, Veronica Calabrò, Valerio Velardi, Bruno Fabris, Stella Bernardi

**Affiliations:** 1 Department of Medical Surgical and Health Sciences, University of Trieste, Cattinara Teaching Hospital Strada di Fiume 449, 34149 Trieste, Italy; margherita.paccagnella@studenti.units.it (M.P.); anna.pizzo@studenti.units.it (A.P.); valerio.velardi@studenti.units.it (V.V.); b.fabris@fmc.units.it (B.F.); 2 Unit Endocrinology Medicina Clinica, ASUGI, Cattinara Teaching Hospital Strada di Fiume 449, 34149 Trieste, Italy; veronica.calabro@asugi.sanita.fvg.it

**Keywords:** thyrotoxicosis, Graves’ disease, cerebral venous thrombosis, case report, mechanisms

## Abstract

Thyrotoxicosis appears to be a predisposing factor for cerebral venous thrombosis (CVT), which is a rare but important cause of stroke in young adults. The presentation of CVT is highly variable, ranging from a history of headaches (in the majority of cases) to deep coma, with the latter requiring invasive neurosurgical decompression. Although the long-term outcomes of CVT are favorable, multicenter cohort studies have shown that death may occur in up to 4% of cases in the acute phase and 8–10% of cases in the long term. It has been argued that the substantial decrease in mortality in patients with CVT that has been observed during the past few decades may be the result of an increased awareness of CVT among clinicians. Given that thyrotoxicosis is a risk factor for CVT, clinicians (and endocrinologists) should be alert to the possibility of CVT in patients with thyroid disease in order to prevent it whenever possible or treat it promptly. In this review, we provide an updated overview of the characteristics of patients with thyrotoxicosis who presented with CVT, the underlying mechanisms, and a few tips for clinicians.

## 1. Introduction

Since the 1990s, when the first cases of thyrotoxicosis with cerebral venous thrombosis (CVT) were described [1,2,3,4,5], thyroid hormone excess has emerged as a predisposing factor for CVT [6], and it appears to be more common than initially thought [7]. Verberne et al. were the first to underline that the incidence of combined thyrotoxicosis and CVT was higher than could be attributed to chance alone [6].

Overall, the literature shows that, on the one hand, patients with thyroid hormone excess are at higher risk of thrombosis, with an odds ratio of 2.2 [8,9,10,11,12,13,14]. For example, in a retrospective cohort study of 428 patients with thyrotoxicosis, 0.7% (C.I. 0.14–2%) of patients (3/428) had a documented episode of thrombosis within 6 months of thyroid disease onset [12]. This rate of thrombosis appeared to be higher than would be expected from general population studies. In particular, one of these patients, a 23-year-old woman taking the oral contraceptive pill (OCP), had a cerebral venous thrombosis [12], in line with the concept that patients with CVT usually have multiple risk factors [15].

On the other hand, when looking at cohorts of patients with cerebral venous thrombosis, thyroid disease is counted as one of its risk factors, with a prevalence ranging from 1.7% [15] to 20.9% [7]. In a prospective multicenter study on 624 CVT patients who were followed for 16 months, thrombophilia and OCP were the most common risk factors, and thyroid disease was reported in 11 patients out of 624 (1.7%) [15]. More recently, in a retrospective single-center analysis of 182 patients hospitalized between 1996 and 2016 for CVT, a history of thyroid dysfunction at the time of hospitalization was observed in 38 patients (20.9%). Nevertheless, the most common risk factors were genetic or acquired thrombophilia and OCP intake [15].

Although the long-term outcomes of CVT are favorable, multicenter cohort studies have shown that death may occur in up to 4% of cases in the acute phase and in 8–10% of cases in the long term [16]. Silvis et al. have argued that the substantial decrease in mortality in patients with CVT that has been observed during recent decades may partly be the result of an increased awareness of CVT among clinicians [16]. Given that thyroid disease is a risk factor for CVT, clinicians (and endocrinologists) should be alert to the possibility of CVT in patients with thyrotoxicosis in order to prevent it whenever possible and/or treat it promptly.

Based on this background, we present a review of the literature to evaluate (i) the features of patients with CVT and thyrotoxicosis; (ii) the mechanisms underlying this association; (iii) and potential tips for clinicians. The review starts with an illustrative case history/case vignette [16,17].

## 2. Illustrative Case History

**Figure 1:**
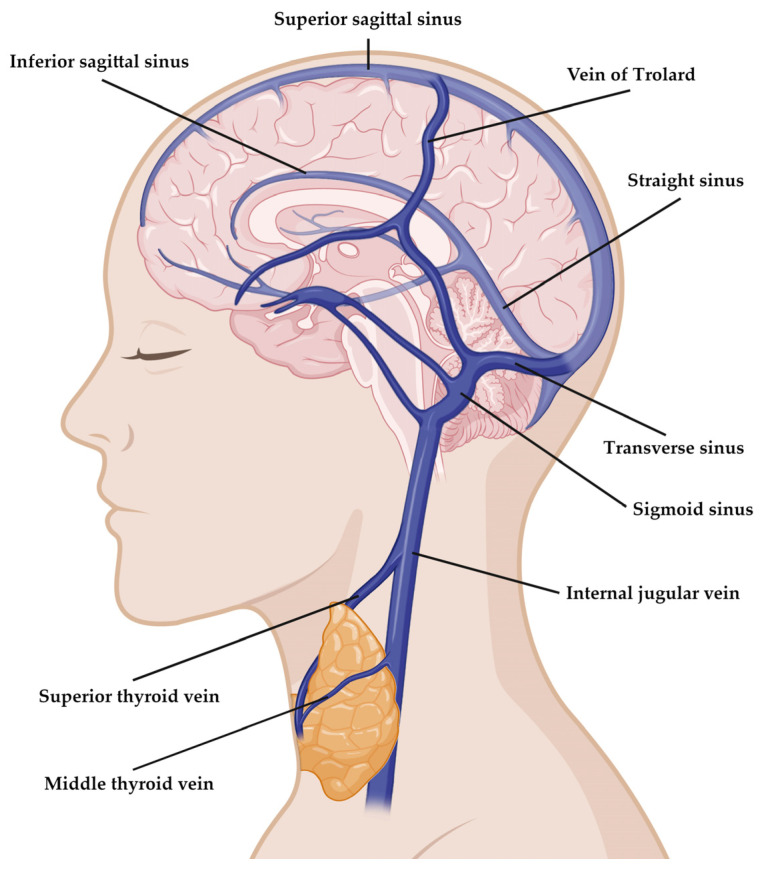
**Anatomy of the cerebral venous system.** The figure shows the sinuses most often involved in CVT among the main components of the cerebral venous system. The sinuses most often involved in patients with thyrotoxicosis and CVT are the superior sagittal sinus, the transverse and sigmoid sinuses, the straight sinus, the internal jugular veins, as well as cortical veins and deep veins [7]. This image was created via Biorender.com.

**Figure 2:**
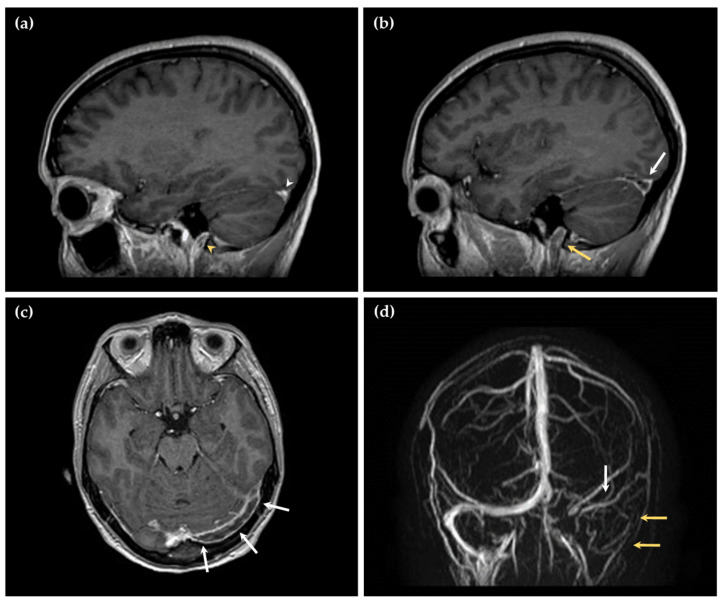
**Magnetic resonance imaging of the illustrative case history**. Sagittal 3D contrast-enhanced T1-weighted images showing (**a**) normal contrast enhancement in the right transverse sinus (white arrowhead) and internal jugular vein (yellow arrowhead); (**b**) a filling defect in contrast enhancement in the left transverse sinus (white arrow) and the left internal jugular vein (yellow arrow), indicating cerebral venous thrombosis; (**c**) axial 3D contrast-enhanced T1-weighted image showing an extensive filling defect in contrast enhancement within the left transverse sinus (white arrows); (**d**) magnetic resonance venography showing no vascular signal on the left transverse and sigmoid sinus (white arrow) extending to the left internal jugular vein (yellow arrows).

A 21-year-old woman presented to the Emergency and Resuscitation service of our hospital with a headache that was unresponsive to medical treatment. According to her medical history, she was a smoker and was taking the OCP. Her blood pressure was recorded as 108/69 mmHg; her heart rate was 133 bpm; and her body temperature was 36.9 °C. Imaging revealed a massive thrombosis of the cerebral venous sinuses, i.e., the transverse sinus, sigmoid sinus, and internal jugular vein on the left hemisphere, with no parenchymal lesions (Figure 1 and Figure 2).

The patient was admitted to the Stroke Unit, and she was treated with low-molecular-weight heparin (LMWH) as the anticoagulant therapy. Due to the presence of tachycardia, her thyroid function was assessed, and exams revealed new-onset thyrotoxicosis due to Graves’ disease (GD). Her testsshowed that TSH was 0.01 microU/mL, free T3 (fT3) was 13 pg/mL, free T4 (fT4) was 52.2 pg/mL, anti-TSH receptor antibodies (ATSHRs) were 9.4 UI/mL, and anti-thyroid peroxidase antibodies were 1203 UI/mL. For this reason, she was prescribed methimazole and propranolol. In addition, the patient was found to have antinuclear antibody (ANA) positivity with a titer of 1:320, as well as weak positivity for lupus anticoagulant (LAC). The test for COVID-19 was negative. One month later, the patient’s thyroid hormone levels normalized, imaging showed an improvement of the vascular defect, and the patient made a complete recovery.

## 3. Features of Patients with Thyrotoxicosis and Cerebral Venous Thrombosis

In order to review the features of patients with thyrotoxicosis and cerebral venous thrombosis, we analyzed studies published from 1991 to June 2024, reporting the cases involving an association between thyrotoxicosis and CVT. This was based on previously published systematic reviews of the literature [18,19], and on a Pubmed search culminating in June 2024. Table 1 reports all the cases of thyrotoxicosis and cerebral venous thrombosis reported in the literature. This includes the patient that we described in our illustrative case history.

In total, we identified 63 described cases of thyrotoxicosis with CVT. The median age was 34 years (the youngest patient was 8 years old; the eldest patient was 68 years old), and the percentage of female sex was 67% (42/63 patients). A total of 49 patients (78%) had thyrotoxicosis due to GD. Thrombophilia (genetic or acquired) was present in 20/63 patients (32%); OCP was present in 14/42 patients (33%). In line with the literature [7,12,62], ethnicity did not appear to affect the risk of CVT in patients with thyrotoxicosis.

These data are consistent with the results of a recent literature update on 39 case reports [19], where patients were mostly young (i.e., <50 years old, 88%) and predominantly female (66%), presenting with headaches (79%). Thyrotoxicosis was attributed to GD in 86% of cases, and 61% of patients had other conditions favoring CVT. The most common condition favoring CVT was thrombophilia, which was present in 30% of patients. This included genetic thrombophilia due to Leiden mutation, protein C and/or protein S deficiency, antithrombin (AT)III deficiency, methylenetetrahydrofolate reductase (MTHFR) mutations, as well as acquired thrombophilia (antiphospholipid syndrome, lupus anticoagulant).

Interestingly, Situmeang et al. have recently reported that mild COVID-19 can precipitate CVT in the presence of thyrotoxicosis [60]. Interestingly, some authors have also found that an association between COVID-19 and CVT is more likely to be seen in older males [63,64], as compared to non-COVID-19 cases of CVT, mostly affecting women in their third decade of life [60]. An updated overview on the association between COVID-19 and CVT can be found in recent systematic reviews [63,65] and observational studies [64].

Nevertheless, from a clinical point of view, the majority of CVT cases presented with severe headache (79%) and involved the superior sagittal sinus (51%), followed by the transverse and sigmoid sinuses. Most importantly, in 81.4% of cases, the outcome was favorable for the patients [19].

It should be noted that the information that can be gathered from case reports may be limited by the fact that some case descriptions might have omitted crucial data, patients did not always undergo the same analyses, and data on the risk of CVT recurrence in these patients were not available, as reviewed by Nissen [66].

## 4. Mechanisms

### 4.1. Anatomy

Thyrotoxicosis has been associated with the risk of venous thrombosis, with an odds ratio (OR) of 2.2 (95%CI 1.0–4.6) [14]. Given that the literature describes the co-occurrence of thyrotoxicosis and CVT in >50 case reports, it seems that beyond the increased risk of deep vein thrombosis [43], thyroid hormone excess is specifically associated with an increased risk of CVT. The precise mechanisms have yet to be elucidated, considering that they are difficult to study due to the very low incidence of CVT [43]. Nevertheless, looking at the anatomy of the cerebral venous system (Figure 1), in some cases of thyroid storm, there might be a mechanical factor contributing to CVT, which is the presence of a goiter reducing the venous outflow from the cranial district (internal jugular vein). In addition, it has to be noted that the superior and middle thyroid veins open into the internal jugular veins with very few variations [67].

### 4.2. Autoimmunity and Inflammation

Thyrotoxicosis might also promote thrombosis and CVT because of autoimmunity and inflammation. Autoimmune diseases are associated with an increased risk of developing venous thrombosis [11] and this risk has been ascribed to the effect of inflammation on coagulation [11]. The most frequent cause of thyrotoxicosis is GD, an autoimmune disease characterized by a nonhomogeneous lymphocytic infiltration of the thyroid gland. Thyrocytes are affected by (auto)immune mechanisms, such as the local production of ATSHR, which stimulates the synthesis and release of thyroid hormones. Patients with GD exhibit not only elevated circulating ATSHR but also high levels of proinflammatory cytokines [68], which may increase the risk of thrombosis.

Interestingly, high levels of proinflammatory cytokines not only result from autoimmunity, in cases of GD, but also from thyroid hormones per se. It has been shown that thyroid hormones regulate the immune system [69]. They induce leukocyte proliferation, migration, release of cytokines (such as interleukin-1), and antibody production, triggering an immune response against either sterile or microbial stimuli [70]. Unsurprisingly, in healthy subjects, there is a positive correlation between thyroid hormones and inflammatory markers, such as IL-6, as well as different subsets of T cells [71].

In addition, many autoimmune diseases may occur concurrently in individuals with a genetic risk of autoimmunity [72]. In particular, GD is associated with an increased risk of systemic lupus erythematosus (SLE), which is a risk factor for CVT [16]. A recent study demonstrated that 16% of patients with GD developed various autoimmune diseases, including SLE [73]. This is due to shared genetic risk factors involving both HLA and non-HLA genes, such as polymorphisms in PTPN22, IFIH1, and ITPR3, which influence the risk of both GD and SLE [72]. Interestingly, it has been shown that antiphospholipid syndrome (APS), which is an autoimmune disease that occurs either as a primary condition or as a part of an underlying disorder, usually SLE, and that manifests as recurrent venous or arterial thrombosis, is also associated with thyroid autoimmunity [74]. This is consistent with earlier studies suggesting that the hypercoagulable status of patients with GD could be sustained by the presence of anti-cardiolipin antibodies, positively identified in over 30% of patients [75].

### 4.3. Coagulation Fibrinolysis and Platelet Function

**Figure 3:**
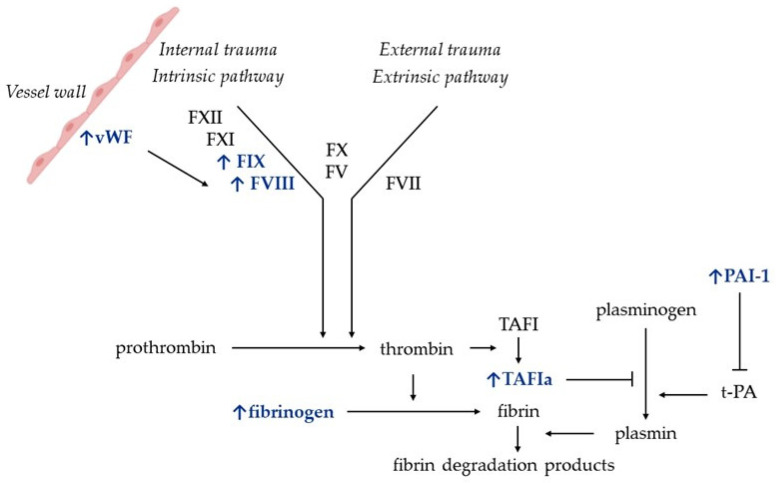
**Schematic representation of coagulation and fibrinolysis pathways and the changes induced by thyroid hormone excess.** The factors that have been identified as having increased in patients with thyrotoxicosis are indicated in blue ink. F, factor; PAI-1, plasminogen activator inhibitor-1; TAFI, thrombin-activatable fibrinolysis inhibitor; TAFIa, activated TAFI; t-PA, tissue-plasminogen activator; vWF, von Willebrand factor. The blue ink shows the factors whose activity increases in case of thyrotoxicosis.

The effect of thyroid hormones on coagulation and the risk of venous thrombosis has been observed in cases of thyrotoxicosis caused by GD, as well as non-autoimmune conditions, which suggests that there must be factors other than inflammation promoting CVT in patients with thyrotoxicosis and that thyroid hormones per se might affect the hemostatic balance. The seminal meta-analysis by Stuijver et al. evaluated the effects of thyroid hormones on the coagulation and fibrinolytic system, and showed that thyrotoxicosis shifts the hemostatic balance towards a hypercoagulable and hypofibrinolytic state with a rise in factors VIII (FVIII) and IX (FIX), fibrinogen, von Willebrand factor (vWF), and plasminogen activator inhibitor-1 (PAI-1), as shown in Figure 3. On the other hand, overt hypothyroidism seems to promote a hypocoagulable state due to reduced levels of coagulation factors [76] and decreased FVIII and vWF activity [77,78]. Notably, it seems that FVIII and vWF are the primary contributors to a hypercoagulable state and venous thrombosis [79]. In line with the hypercoagulable state induced by thyroid hormones, a recent mendelian randomization study showed that genetically predicted hyperthyroidism was associated with increased FVIII and vWF [80]. Also, levothyroxine-suppressive treatment has been associated with significant increases in FVIII, vWF, fibrinogen, and PAI-1 [81]. These changes are in line with the findings of most case reports, indicating that there was increased FVIII activity in most patients with thyrotoxicosis and CVT, as shown in Table 1. In addition, a recent prospective study on 200 patients (64 hyperthyroid, 68 hypothyroid, and 68 euthyroid patients) demonstrated that high levels of FVIII, vWF, and fibrinogen contributed to the hypercoagulable state of hyperthyroid patients, where the rate of thromboembolic manifestations was 6.25%, and increased to 8.3% in cases where the FVIII value was ≥1.5 U/mL [82].

With respect to fibrinolysis, it has been shown that clots in patients with hyperthyroidism show a much denser fibrin network as well as increased clot lysis times. This is due to several mechanisms, the first being PAI-1, which inhibits fibrinolysis—levels of which are increased during thyrotoxicosis [83]. The second is thrombin-activatable fibrinolysis inhibitor (TAFI). Thyroid hormones promote the activation/activity of TAFI. This is a glycoprotein that links the coagulation and fibrinolytic system. It is synthesized in the liver, and circulates as a proenzyme which is activated by thrombin to TAFIa. Once it is activated, it inhibits plasmin formation and fibrin degradation. Verkleij et al. evaluated the effect of hyperthyroxinemia and hypothyroidism on TAFIa and found that exogenous thyroid hormone excess increased TAFIa-dependent prolongation of clot lysis and reduced fibrinolysis, while hypothyroidism had the opposite effect [84].

Most of the effects of thyroid hormones on the coagulation and fibrinolysis systems are mediated by the thyroid hormone receptor β (THRβ), which is widely expressed in the liver, as well as in endothelial cells, which are the source of production of FVIII, FIX and vWF. This has been demonstrated by comparing the parameters of coagulation and fibrinolysis in patients with thyroid hormone excess due to hyperthyroidism vs. defective thyroid hormone receptor β (THRβ). The results indicate that hyperthyroid patients exhibit higher levels of FVIII, fibrinogen, and vWF than those with defective THRβ [85]. Burggraaf et al. found that thyrotoxicosis increased the plasma levels of most endothelial marker proteins, such as vWF, tissue plasminogen activator (t-PA), PAI-1, and thrombomodulin, and of some liver-synthetized proteins [86], highlighting that in cases of thyroid hormone excess, there is also an endothelial activation, which might contribute to the thrombotic risk.

Thyroid hormones have genomic as well as nongenomic actions. Genomic actions are mediated by THRα or THRβ, while nongenomic actions are mediated by direct interaction of fT4 with integrin αvβ3 (also known as vitronectin receptor). The latter appears to be the mechanism whereby high fT4 levels lead to pathologic platelet aggregation, directly or indirectly, via endothelial cells, which increases the risk of coagulation [87].

## 5. Tips for Clinicians

- CVT should be suspected in cases of severe headache, which might be associated with other symptoms of intracranial hypertension such as nausea, papilloedema, decreased visual activity, and tinnitus [15]. Other manifestations include acute symptomatic seizures and—less frequently—focal motor deficit, aphasia, mental status disorders, movement disorder, and coma [15]. Computed tomography or magnetic resonance venography are the imaging modalities for diagnosis of CVT, while magnetic resonance is the best modality for detecting brain parenchymal lesions. Low-molecular-weight heparin is generally preferred as a first-line treatment over unfractionated heparin [15].
- The exclusion of thyrotoxicosis should be considered in all patients with suspected CVT, including when other risk factors for CVT are present [7,19]. This is not only because any underlying condition that might have contributed to the disease should be corrected if possible, but also because thyrotoxicosis could significantly impact on patients’ clinical condition and affect the outcome [17,56]. On the other hand, given that CVT often occurs due to multifactorial hypercoagulability, the presence of thyrotoxicosis does not rule out the presence of other risk factors. Therefore, patients with CVT and thyrotoxicosis should undergo a thorough assessment of any other risk factors for thrombosis, such as genetic and/or acquired thrombophilia, as well as sex-specific factors (e.g., oral contraceptives, pregnancy) and other disease (e.g., cancer).
- Thyrotoxicosis should be considered as a minor (yet important) transient risk factor for thrombosis, as high levels of fT4 combine with other stimuli to cross the so-called “thrombotic threshold”, mainly by increasing FVIII and vWF [43]. There is no indication for thrombophylactic treatment in patients with thyrotoxicosis [43]. Nevertheless, smokers should be encouraged to quit, and caution should be taken when prescribing drugs that increase the risk of CVT, such as OCP in the acute phase of thyrotoxicosis.
- The risk of recurrence of CVT is generally low [88,89,90]. Previous studies have shown that the recurrence rate of CVT is between 0.53 and 1.5 per 100 person-years [88,89]. Recurrences occur more often in the first year and among men [91]. Thyrotoxicosis does not seem to be associated with a higher risk of recurrence.

## 6. Conclusions

Thyrotoxicosis and CVT are associated with a higher incidence than expected by chance alone. Clinicians (and endocrinologists) should be alert to the possibility of CVT in patients with thyrotoxicosis, and should thus consider thyrotoxicosis in cases of CVT. This association has been ascribed to anatomy, autoimmunity and inflammation, as well as direct effects of thyroid hormones on coagulation, fibrinolysis, and platelets. Based on >50 case reports, the literature shows that this association affects mostly young women with GD and is often accompanied by other risk factors, such as OCP or thrombophilia, and that it should be suspected in cases of severe headache. Computed tomography or magnetic resonance are the preferred imaging modalities, and heparin is the treatment of choice. Long-term outcomes are favorable, and the substantial decrease in mortality that has been observed over the past few decades may be attributed to an increased awareness of CVT among clinicians [16].

The limitations of the currently available evidence on this topic hamper the formulation of specific recommendations for clinical practice. For this reason, further studies should include thyroid function as a continuous variable in prediction studies on thrombosis and CVT risk; conduct prospective cohort studies in patients with biochemically diagnosed thyrotoxicosis vs. patients with normal thyroid function; and address the safety and effectiveness of thrombophylactic therapy in high-risk patients with thyrotoxicosis.

## Figures and Tables

**Table 1:** Cases of thyrotoxicosis associated with thrombosis.

Ref	Age	Sex	Graves’ Disease	Thrombophilia	OCP	Site	Treatment
Schutta 1991 [1]	34	M	?	↓Plasminogen	NA	SSS; IJV	PTU VKA
Siegert 1995 [2]	24	M	?	No	NA	SSS	Steroid therapy VKA
Siegert 1995 [2]	32	F	?	No	No	SSS; TS	PTU steroid therapy
Silburn 1996 [3]	18	F	Yes	No	Yes	DCV; ISS	Anticoagulants
Dulli 1996 [4]	32	F	?	↓Protein C	Yes	SSS; TS	RAI UFH VKA
De Scryver 1999 [5]	32	M	Yes	LAC	NA	TS + SiS	MMI LT4 LMWH
Dai 2000 [20]	39	M	?	No	NA	SSS	MMI LMWH ASA
Longe 2000 [21]	38	M	Yes	No	NA	SSS	MMI steroid therapy
Longe 2000 [21]	18	M	No	No	NA	SSS	MMI steroid therapy UFH VKA
Verberne 2000 [6]	28	F	Yes	No	Yes	SS; TS + SiS; IJV	MMI KI LMWH
Ra 2001 [22]	60	M	?	No	NA	SSS; SiS	MMI urokinase
Maes 2002 [23]	39	F	Yes	↑FVIII	Yes	TS; IJV	MMI UFH VKA
Colleran 2003 [24]	37	F	Yes	↑homocysteine ANA	?	CVT	MMI
Molloy 2003 [25]	28	F	Yes	FV Leiden mutation	?	CVT	Anticoagulants
Karouache 2004 [26]	38	F	Yes	No	No	TS + SiS	MMI steroid therapy heparin
Karouache 2004 [26]	44	F	Yes	No	?	SSS; TS + SiS	Steroid therapy heparin
Madronero-Vuelta 2004 [27]	42	F	No	FII mutation	?	SSS; TS	Anticoagulants
Mounton 2005 [28]	32	F	No	↑FVIII	Yes	TS	UFH
Mounton 2005 [28]	49	F	Yes	↑FVIII ↓Protein C	No	SSS; TS	UFH
Mounton 2005 [28]	50	F	Yes	↑FVIII	Yes	TS Cortical veins	?
Mounton 2005 [28]	38	F	Yes	↑FVIII	Yes	SSS; TS	?
Kasuga 2006 [29]	39	M	Yes	↑FVIII	NA	SSS; TS	MMI UFH VKA
Nagumo 2007 [30]	28	F	Yes	↑FVIII ↓Protein C	?	SSS	No (died)
Pekdemir 2008 [31]	28	M	No	No	NA	TS + SiS	UFH
Strada 2008 [32]	29	M	Yes	MTHFR mutation	NA	SSS; TS	MMI UFH
Usami 2009 [33]	34	F	Yes	↑FVIII ↑vWF ↓Protein C	No	SS; SSS; TS Cortical veins	MMI UFH VKA
Bensalah 2011 [34]	23	M	Yes	Steroid therapy	NA	SSS; TS + SiS	LMWH VKA
Hermans 2011 [35]	22	F	Yes	No	Yes	SiS	MMI steroid therapy UFH
Hwang 2012 [36]	31	M	No	↑FIX; ↑FXI	NA	SSS	PTU VKA
van Eimeren 2012 [37]	8	F	Yes	↑FII; ↑FVIII, ↑FIX; ↑FXI FV mutation	No	Cerebral sinus veins	MMI LMWH UFH
Aggarwal 2013 [38]	44	F	No	No	No	SS; SSS	LMWH VKA
Chhabra 2013 [39]	45	F	?	↑FVIII	No	SSS; TS	MMI I^-^ LMWH
Janovsky 2013 [40]	21	F	Yes	↑FVIII APLA	No	SSS; TS + SiS	MMI craniotomy LMWH
Kim 2013 [12]	23	F	Yes	?	Yes	CVT	MMI anticoagulant
Migeot 2013 [41]	26	F	Yes	↑FVIII	No	SSS; TS	MMI VKA
Srikant 2013 [42]	42	F	Yes	↓Protein S ↓ATIII	No	SS; TS	MMI craniotomy LMWH ASA
Elbers 2014 [43]	50	F	Yes	No	No	TS + SiS	PTU KI LMWH VKA
Knudsen-Baas 2014 [19]	17	F	Yes	?	Yes	TS + SiS; IJV	MMI LMWH
Liu 2015 [44]	44	F	Yes	↑FVIII ↓ATIII	Yes	SS; TS + SiS	PTU craniotomy LMWH VKA
Hieber 2016 [19]	52	F	Yes	↓Protein C	No	TS + SiS	MMI LMWH VKA
Kim 2016 [19]	39	M	Yes	↓Protein C ↓Protein S	NA	SSS	MMI UFH VKA
Waheed 2016 [45]	48	F	Yes	↑FVIII; ↑vWF; RF; ANA; pANCA	Yes	DCV SS	PTU UFH VKA
Fu 2017 [46]	45	M	Yes	↑FVIII	NA	TS	LMWH VKA
Grewal 2017 [47]	22	M	Yes	↑FVIII	NA	SSS; TS + SiS	UFH; MMI
Kawahara 2017 [48]	68	F	Yes	?	No	SSS; TS	Anticoagulant
Kraut 2017	62	F	Yes	↑FVIII	No	SSS; TS + SiS; IJV	MMI UFH VKA
Tanabe 2017 [49]	49	F	Yes	?	?	TS + SiS; IJV	MMI KI steroid therapy UFH thrombectomy
Madan 2018 [50]	28	F	Yes	?	No	TS + SiS	MMI steroid therapy LMWH VKA
Rehman 2018 [51]	31	M	Yes	LAC	NA	SS; SSS; TS	MMI I^-^ LMWH VKA
Son 2019 [52]	31	M	Yes	?	NA	SSS; TS + SiS	LMWH
Yokoyama 2019 [53]	48	F	?	↑FVIII	No	SSS; common femoral vein	I^-^ UFH VKA
Chee 2020 [54]	40	F	Yes	?	No	TS + SiS; IJV	MMI craniotomy LMWH NOAC
Elhassan 2020 [55]	41	M	Yes	No	NA	SSS Cortical veins	MMI LMWH NOAC
Anuszkiewicz 2021 [56]	15	M	Yes	↑FVIII; ↑vWF	NA	SSS; TS + SiS	MMI UFH
Gomes 2021	23	F	Yes	?	No	SS;TS + SiS	MMI LMWH VKA
Fandler-Hofler 2022 [57]	60	F	Yes	?	No	TS + SiS	MMI LMWH NOAC
Fandler-Hofler 2022 [57]	33	F	Yes	?	Yes	SS; TS; IJV	LMWH MMI
Gong 2022 [58]	29	M	Yes	↓Protein C	NA	SS; SSS; TS	MMI PTU I^-^ steroid therapy plasmapheresis LMWH NOAC
Jia 2022 [59]	32	F	Yes	?	?	SS; SSS; TS + SiS	MMI LMWH urokinase alteplase thrombus aspiration
Situmeang 2022 [60]	37	M	Yes	No	NA	SSS; TrV; TS + Sis	MMI LMWH NOAC
Raho 2023 [19]	46	F	Yes	No	No	SS Confluence of sinuses	MMI PTU LMWH VKA
Tashiro 2023 [61]	38	F	Yes	↑FVIII ↑vWF	No	SSS; TS + SiS	PTU UFH NOAC
Our case	21	F	Yes	ANA LAC	Yes	TS + SiS; IJV	MMI UFH VKA

ANA, antinuclear antibody; APLA, antiphospholipid antibodies; ASA, acetylsalicylic acid; ATIII, antithrombin III; CVT, cerebral venous thrombosis; DCV, deep cerebral veins; F, factor; I, iodide; IJV, internal jugular vein; ISS, inferior sagittal sinus; KI, potassium iodide; LAC, lupus anticoagulant Ab; LMWH, low-molecular-weight heparin; LT4, levothyroxine; MMI, methimazole/carbimazole; thiamazole; MTHFR, methylenetetrahydrofolate reductase; NA, not applicable (in males); NOAC, non-vitamin K antagonist oral anticoagulants; OCP, oral contraceptive pill; pANCA, perinuclear antineutrophil cytoplasmic antibody; PTU, propylthiouracil; RAI, radioiodine therapy; RF, rheumatoid factor; SiS, sigmoid sinus; SS, straight sinus; SSS, superior sagittal sinus; TrV, trolard vein; TS, transverse sinus; UFH, unfractionated heparin; VKA, vitamin K antagonists; vWF, von Willebrand factor; “?”, not specified; “↑”, increased levels. ↓ decreased levels.

## Data Availability

Not applicable.
